# Emergency Physician Performed Ultrasound-Guided Abdominal Paracentesis: A Retrospective Analysis

**DOI:** 10.24908/pocus.v9i1.16668

**Published:** 2024-04-22

**Authors:** Brandon M Wubben, Jad Dandashi, Omar Rizvi, Srikar Adhikari

**Affiliations:** 1 Department of Emergency Medicine, University of Arizona College of Medicine Tucson, AZ USA; 2 Department of Emergency Medicine, University of Iowa Carver College of Medicine Iowa City, IA USA

**Keywords:** Emergency Ultrasound, Procedures, Point-of-care-ultrasound (POCUS), Ultrasound Guided Procedures, Parecentesis, Emergency medicine

## Abstract

Background: Emergency physicians commonly perform ultrasound-assisted abdominal paracentesis, using point of care ultrasound (POCUS) to identify ascites and select a site for needle insertion. However, ultrasound-guided paracentesis has the benefit of real-time needle visualization during the entire procedure. Our objective was to characterize the performance of emergency physician-performed ultrasound-guided paracentesis using POCUS, their ability to achieve good in-plane needle visualization, and factors associated with procedural success. Methods: A POCUS database was retrospectively reviewed for examinations where abdominal paracentesis was performed by an emergency physician at two academic urban emergency departments over a six-year period. Medical records were reviewed for demographics, presenting history, complications, and hospital course. Descriptive statistics were used to summarize the data. Results: 131 patients were included in the final analysis. The success rate for ultrasound-guided paracentesis was 97.7% (84/86 [95% CI: 92-100%]) compared to 95.6% (43/45 [95% CI: 85-99%]) for ultrasound-assisted paracentesis (p=0.503). 58% (50/86) demonstrated good in-plane needle visualization; 17% (15/86) had partial or out-of-plane visualization; and 24% (21/86) did not demonstrate needle visibility on their saved POCUS images. All four procedural failures were performed by first- or second-year residents using a curvilinear transducer, while all procedures using a linear transducer were successful. The most common complications were ascites leak, infection at the site, and minor bleeding. Conclusions: Emergency physicians with training in real-time needle guidance with ultrasound were able to use POCUS to perform ultrasound-guided paracentesis in the emergency department with a high success rate and no fatal complications. Based on our experience, we recommend performing ultrasound-guided paracentesis using a linear transducer, with attention to identifying vessels near the procedure site and maintaining sterile technique.

## Background

Diagnostic abdominal paracentesis is an essential emergency department (ED) procedure. Emergency physicians commonly employ ultrasound-assisted paracentesis, in which point of care ultrasound (POCUS) is used to identify ascites and mark an ideal location for needle entry.^1^ Ultrasound-assisted paracentesis has been shown to be more successful and result in fewer complications than a landmark-based approach, but requires an adequately-sized volume of ascites to be performed safely 1, 2, 3, 4, 5.

For smaller volumes of ascites, ultrasound-guided paracentesis, in which ultrasound is used for real-time needle visualization during the entire procedure, may be utilized 4. Ultrasound-guided paracentesis has been shown to be successful with minimal complications when performed by radiologists 6. However, waiting for interventional radiology to perform paracentesis is associated with delays in care 7. Emergency physicians already use real-time ultrasound needle guidance for other emergent procedures, such as vascular access 8. To our knowledge, the ability of emergency physicians to use POCUS for real-time needle guidance during paracentesis has not been previously studied.

The objective of this study was to evaluate (1) the success and complication rates of ultrasound-guided paracentesis compared to ultrasound-assisted paracentesis in the ED, (2) the ability of emergency physicians to achieve real-time in-plane POCUS needle guidance, and (3) the procedure and patient-related factors associated with success.

## Methods

### Study Design/Study Setting

A retrospective review of ED patients who received paracentesis using ultrasound in the ED at two academic urban hospitals with a total annual census of 125,000 between July 2014 and October 2021 was performed. These hospitals support two ACGME (Accreditation Council for Graduate Medical Education) emergency medicine residency programs – one combined emergency medicine and pediatrics residency, and one emergency ultrasound fellowship that operates at both hospitals. 

Emergency medicine residents typically have minimal prior ultrasound experience at the beginning of their residency training. All emergency medicine residents receive real-time needle guidance training for performing procedures as part of their internal orientation, led by residency leadership. Additionally, during the ultrasound orientation day, emergency medicine residents participate in didactic and hands-on training sessions on needle guidance using phantoms. All emergency medicine first year emergency medicine residents undergo a two-week emergency ultrasound rotation, gaining both didactic instruction and supervised scanning experience in the ED. This includes specific training on real-time needle guidance for procedures. They are required to review materials on ultrasound-guided procedures and take quizzes as part of the evaluation process. They also receive direct instruction on needle guidance while performing procedures in the ED during this two-week rotation. Furthermore, residents attend recurring didactic sessions, with at least three sessions annually focused on performing ultrasound-guided procedures, such as paracentesis. Finally, they participate in hands-on training in various ultrasound-guided procedures that require real-time needle guidance, offered four times a year during simulation training sessions.

Multiple ultrasound systems were available for use in the ED, including Mindray M9 (Shen Zhen, China), Philips CX50 (Amsterdam, The Netherlands), Zonare ZS3 (Zonare Medical Systems, Mountain View, California), and Philips Sparq (Philips Healthcare, Andover, Maryland). All systems are equipped with a curvilinear and a high frequency linear transducer. All POCUS images acquired in the ED are archived in the web-based middle-ware Q-path (Q-path, Telexy Healthcare, BC, Canada) and undergo quality assurance by an emergency ultrasound fellow or faculty. 

### Study Protocol

The Q-path ultrasound image archiving system (Telexy Healthcare, Maple Ridge, BC, Canada) was queried for eligible patients who underwent abdominal paracentesis using POCUS in the ED. Patients were excluded for several reasons. These include if there was no corresponding documentation available for the procedure in the electronic medical record; the procedure was not performed by an ED provider; the procedure was not performed due to minimal or no accessible fluid pocket, or patient refusal as documented by the performing physician; the examination was a duplicate from the same procedure; or the Q-path record was incorrectly labeled as a paracentesis. 

First, one chart reviewer performed electronic medical record review and data abstraction using a standardized data extraction form via REDCap 9, including patient demographic characteristics, relevant labs, documented success of the procedure, number of attempts, volume of fluid aspirated, disposition, and admitting or discharge diagnosis. The discharge summary (if admitted) or ED document (if discharged) were reviewed. Second, ultrasound images in the Q-path database were reviewed by two emergency ultrasound fellowship-trained physicians to determine the level of training of the performing physician, type of transducer utilized, depth of the fluid pocket relative to peritoneum based on on-screen depth ruler, the technique attempted (ultrasound-guided versus assisted, as documented in the Q-path procedure note), identification of inferior epigastric vessels (considered attempted if a Doppler box was used, and visualized if there was Doppler captured pulsatile flow in the expected location), and whether in-plane or out-of-plane visualization was achieved. The primary author of the procedural documentation was assumed to be the performing provider. However, because all residents are supervised by an attending physician in the ED, the supervising physician could have aided in some cases. The reviewers independently rated image quality on a Likert scale from 1 (uninterpretable images) to 5 (excellent) with 3 (minimum criteria for diagnosis) as a median. Depth measurements and quality ratings were averaged between reviewers. Statistical analysis was performed using SPSS (IBM SPSS Statistics for Macintosh, Version 28. Armonk, NY: IBM Corp). Continuous data are presented as means, and dichotomous data are presented as percent frequencies of occurrence with 95% confidence intervals (CIs). The statistical level of significance used in all analyses was 0.05. This study was approved by the Institutional Review Board. 

## Results

A total of 131 patients who had abdominal paracentesis performed in the ED using POCUS were included in the final analysis. Thirty-five patients were excluded. Patient and procedure characteristics are shown in Table 1. 

**Table 1:** Comparison of patient and procedure characteristics between patients undergoing ultrasound-assisted and ultrasound-guided paracentesis in the emergency department using point of care ultrasound.

**Characteristics**	**Ultrasound-Assisted (n=45)**	**Ultrasound-Guided (n=86)**	**p-value**
Age, years (SD)	54.5 (±12.1)	55.1 (±11.6)	0.789
**Sex**			0.657
Female	18 (40%)	31 (36%)	
Male	27 (60%)	55 (64%)	
Body Mass Index	27.1 (±6.0)	27.8 (±6.4)	0.653
**Labs**			
Hemoglobin (SD)	10.8 (±2.1)	10.5 (±2.4)	0.478
Platelets (SD)	182.3 (±130.2)	169.7 (±103.7)	0.556
International Normalized Ratio (SD)	1.6 (±0.5)	1.8 (±0.7)	0.131
**Ultrasound Data**			
Average Skin Thickness, cm (SD)	1.8 (±0.7)	1.7 (±0.7)	0.448
Average Ascites Depth, cm (SD)	5.6 (±2.1)	4.9 (2.6)	0.168
Volume Aspirated, mL (SD)	2306 (±2375)	1928 (±2313)	0.388
Procedure Intent			0.470
Diagnostic	17 (37.8%)	41 (47.7%)	
Therapeutic	5 (11.1%)	4 (4.7%)	
Both	22 (48.9%)	39 (45.3%)	
Procedure Location			0.238
Right Lower Quadrant	30 (66.7%)	63 (73.3%)	
Midline	1 (2.2%)	0 (0%)	
Left Lower Quadrant	12 (26.7%)	15 (17.4%)	
Diagnosis			0.322
Uncomplicated Ascites	31 (68.9%)	66 (76.7%)	
Spontaneous Bacterial Peritonitis	5 (11.1%)	11 (12.8%)	
Unknown/Other	9 (20.0%)	9 (10.5%)	

There were no significant differences in triage vitals or the rate of hospital admission (68.9 vs. 66.3%) between ultrasound-assisted and ultrasound-guided groups. The observed success rate for ultrasound-guided paracentesis was 97.7% (84/86 [95% CI: 92-100%]) compared to 95.6% (43/45 [95% CI: 85-99%]) for ultrasound-assisted paracentesis (p=0.503); procedural outcomes are summarized in Table 2. The depth of ascites fluid was not significantly smaller for failures versus successes (6.1±2.4 vs 5.1±2.5 cm, p=0.45).

**Table 2:** Comparison of outcomes between patients undergoing ultrasound-assisted and ultrasound-guided paracentesis in the emergency department using point of care ultrasound.

**Outcome**	**Ultrasound-Assisted (n=45)**	**Ultrasound-Guided (n=86)**	**p-value**
Procedure Successful (%)	43 (95.6)	84 (97.7)	0.503
Inferior Epigastric Vessels (%)			0.015
Visualized	0 (0)	7 (8.1)	
Attempted	4 (9.8)	19 (22.1)	
Not Shown	41 (90.2)	60 (69.8)	
**Complications (%)^a^**			
Severe Pain	1 (2.2)	0	0.165
Minor Bleeding	1 (2.2)	1 (1.2)	0.639
Major Bleeding	0	1 (1.2)	0.468
Infection	0	3 (3.5)	0.205
Ascites Leak	0	5 (5.8)	0.099
Death	0	0	-
a = One patient in the ultrasound-guided group had both ascites leak and infection.			

Conservatively, including complications theoretically attributable to paracentesis, the proportion of patients experiencing complications was 10.5% (9/86) with ultrasound-guided paracentesis and 4.4% (2/45) with ultrasound-assisted paracentesis (p=0.238). The complications in the ultrasound-assisted patients were minor. One patient reported persistent pain at the paracentesis site and one patient had minor superficial bleeding that resolved with direct pressure. The complications in the ultrasound-guided patients included five patients with leaking ascites and one patient with minor superficial bleeding that resolved with a pressure dressing. One patient developed an abdominal wall hematoma that required two red blood cell transfusions. It was unclear if this was secondary to the ED paracentesis or the interventional radiology-performed paracentesis shortly thereafter and was not attributed to an inferior epigastric vessel injury. Two patients developed abdominal wall cellulitis, including one that required intravenous antibiotics and one that required incision and drainage of an associated abscess followed by operative surgical debridement of the abdominal wall. Another patient developed Staphylococcus epidermidis bacteremia which was included as a possible complication due to unknown source.

For real time ultrasound-guided paracentesis, 58% (50/86) demonstrated good in-plane needle visualization (needle tip and shaft fully seen) and 17% (15/86) had partial or out-of-plane visualization. Twenty four percent (21/86) did not demonstrate needle visibility on their saved POCUS images despite documenting use of real-time guidance. The average image quality was rated as 3.5 (SD±0.5) in the ultrasound-assisted group compared to 3.6 (SD±0.8) in the ultrasound-guided group (p=0.292). There was moderate or better agreement between the two raters with respect to image quality (ICC=0.62, [95%CI: 0.47-0.73]), measurement of ascites depth (ICC=0.79, [95%CI: 0.69-0.85]), and measurement of skin thickness (ICC=0.82 [95%CI: 0.74-0.87]). 

The overall success rate was 97.6% (40/41) for first year residents, 91.4% (32/35) for second year residents, and 100% (55/55) for third-fifth year residents, fellows, and faculty. All four unsuccessful procedures occurred while using a curvilinear transducer (4/87), and there were no unsuccessful procedures (0/44) using a linear transducer (p=0.149). Patients had a higher average BMI in the curvilinear group than the linear group (28.6±6.3 vs. 24.9±5.3, p=0.017). 

## Discussion

We found that emergency medicine physicians were highly successful at performing ultrasound-guided paracentesis in the ED using POCUS. The success rate for ultrasound-assisted paracentesis (96%) was similar to a prior randomized control trial of ultrasound-assisted paracentesis including emergency medicine residents (95%) 1. To our knowledge, the current study is the first to report success rates for emergency medicine physicians performing ultrasound-guided paracentesis (98%).

The types of complications we observed for ultrasound-guided paracentesis – such as bleeding, infection, and ascites leak – were similar to prior studies of paracentesis performed outside of the ED. Abdominal wall infection occurred in 2.3% (2/86) of observed patients. A prior study of inpatient paracentesis using ultrasound found 0.41% (3/723) had infectious complications, but they did not differentiate between ultrasound-assisted and ultrasound-guided procedures, and a similar percentage of infectious complications (2.4%, 14/574) were observed with no use of ultrasound at all.^3^ After receiving both an ED-performed and interventional radiology-performed paracentesis, one patient (1.2%) required red blood cell (RBC) transfusion; a prior retrospective study of 3,116 ultrasound-guided paracenteses performed by radiologists observed hemorrhage requiring RBC’s or angiogram in 0.19% 6. The most common complication was ascites leak (5.8%, 5/86), similar to the 5.0% leak rate reported in a prospective study mostly without using ultrasound 10. We agree that performing the recommended “z-track” technique (where the non-dominant hand is used to put tension on the skin during puncture to decrease post-procedure leaking) might be more difficult when that hand is also holding the ultrasound probe 4, 8. However, many of the complications we observed seem unlikely to be directly attributable to the use of real-time ultrasound guidance, and may instead be associated with patient factors like a smaller ascites volume. The average ascites sampling area was numerically smaller for patients who had real-time guidance, although not statistically different in size. It is possible that real-time guidance in these patients was still safer than it would have been if ultrasound-assisted paracentesis had been performed due to unmeasured patient-level factors. Future prospective study may be needed to identify factors contributing to the demonstrated complications.

The number of residents who achieved good in-plane needle visualization on their saved clips despite attempting an ultrasound-guided technique was low (58%). It is uncertain to what degree this was secondary to lack of provider skill versus non-procedural factors, such as timing of image capture by an assistant, but was similar to the 51% of residents achieving good needle visualization in a study of ultrasound-guided arthrocentesis 11. Overall, in 75% of cases real-time needle visualization (complete and partial) was demonstrated. However, retrospective review of ultrasound images has limitations for determining residents' ability to achieve good in-plane needle visualization with real-time ultrasound-guided procedures. Future research could include prospective evaluation of needle-guidance quality to identify specific targets for interventions to improve needle visualization during ultrasound-guided procedures, better quantify the learning curve of this important skill, and investigate ways to further decrease complications. 

The only unsuccessful procedures in our study were performed by first and second year residents, all using a curvilinear probe. The higher success rate for third year residents and above is similar to that of ultrasound-guided arthrocentesis in the ED, suggesting a learning curve 11. Previously, the use of a linear transducer has been suggested for thin patients and phased-array for larger patients 4. However, real-time needle tracking is easier with a linear transducer since the area of interest (ascites) is superficial, generally within 5 cm. A linear transducer emits only parallel ultrasound beams and allows for the same resolution in the near-field and the far-field, making it ideal for needle tracking as the needle is advanced from the skin into the abdomen. We recommend using a curvilinear transducer for confirming the presence of ascites and choosing the ideal location to perform the procedure, then switching to linear array transducer to perform the procedure. The linear array transducer provides the added benefit that it can be used to visualize the inferior epigastric artery (IEA) prior to needle insertion, as damage to the IEA can cause significant morbidity and mortality 12, 13. 

### Limitations

This study has several inherent limitations due to its retrospective nature. First, only cases where ultrasound images were saved were included. While it is standard practice at our institution to save all images used for patient care, some physicians may have chosen not to perform paracentesis on patients who they felt were likely to be too technically challenging based on initial examination without saving any images, spuriously improving success rates due to selection bias. Additionally, physicians with higher skill levels could have been more likely to save images. There may have been patient-specific factors which led a physician to choose a particular approach where the alternate may have been less successful. However, a wide range of ascites fluid pocket sizes, body mass index (BMI), and skin thickness were included. Finally, while not all data collectors were blinded to the study hypothesis, we attempted to minimize the bias in retrospective data collection by using a standardized abstraction form.

## Conclusions

Emergency medicine physicians with training in real-time needle guidance with ultrasound were able to use POCUS to perform ultrasound-guided paracentesis in the ED with a high rate of success, similar to the success rates observed for ultrasound-assisted paracentesis. Improving in-plane needle visualization remains an educational goal. Based on our experience, we recommend performing ultrasound-guided paracentesis using a linear transducer, with attention to identifying vasculature near the procedure site and maintaining sterile technique.

## Disclosures

The authors disclose the following actual or potential conflicts of interest within the last 36 months: BW, JD, OR have nothing to disclose; SA, funding from the US NIH and Department of Defense, Springer Book Royalties, Consulting Fees from GE and Exo Ultrasound, Honraria from the American Society of Regional Anesthesia and Pain Medicine, and board membership to the American Institute of Ultrasound in Medicine. 
